# Therapeutic Benefit in Rheumatoid Cachexia Illustrated Using a Novel Primary Human Triple Cell Coculture Model

**DOI:** 10.1155/2022/1524913

**Published:** 2022-06-02

**Authors:** Tracey Ollewagen, Gareth S. Tarr, Kathryn H. Myburgh, Helmuth Reuter, Carine Smith

**Affiliations:** ^1^Dept Physiological Sciences, Science Faculty, Stellenbosch University, Stellenbosch, South Africa; ^2^Winelands Rheumatology Centre, Stellenbosch, South Africa; ^3^Dept of Medicine, Faculty of Medicine and Health Sciences, Stellenbosch University, Stellenbosch, South Africa; ^4^Division of Clinical Pharmacology, Dept of Medicine, Faculty of Medicine and Health Sciences, Stellenbosch University, Stellenbosch, South Africa

## Abstract

**Background:**

The loss of muscle mass in rheumatoid arthritis (RA), termed rheumatoid cachexia, is predicted to result from the complex interactions between different cell types involved in the maintenance of skeletal muscle mass, namely, myoblasts, fibroblasts, and macrophages. The complexity within the muscle is further highlighted by the incidence of nonresponsiveness to current RA treatment strategies.

**Method:**

This study aimed at determining differences in the cellular responses in a novel human primary cell triple coculture model exposed to serum collected from nonarthritic controls (NC), RA treatment naïve (RATN), and RA treatment-nonresponding (RATNR) patients. Bone morphogenetic protein-7 (BMP-7) was investigated as a treatment option.

**Results:**

Plasma analysis indicated that samples were indeed representative of healthy and RA patients—notably, the RATNR patients additionally exhibited dysregulated IL-6/IL-10 correlations. Coculture exposure to serum from RATNR patients demonstrated increased cellular growth (*p* < 0.001), while both hepatocyte growth factor (*p* < 0.01) and follistatin (*p* < 0.001) were reduced when compared to NC. Furthermore, decreased concentration of markers of extracellular matrix formation, transforming growth factor-*β* (TGF-*β*; *p* < 0.05) and fibronectin (*p* < 0.001), but increased collagen IV (*p* < 0.01) was observed following RATNR serum exposure. Under healthy conditions, BMP-7 exhibited potentially beneficial results in reducing fibrosis-generating TGF-*β* (*p* < 0.05) and fibronectin (*p* < 0.05). BMP-7 further exhibited protective potential in the RA groups through reversing the aberrant tendencies observed especially in the RATNR serum-exposed group.

**Conclusion:**

Exposure of the triple coculture to RATN and RATNR serum resulted in dysregulated myoblast proliferation and growth, and ECM impairment, which was reversed by BMP-7 treatment.

## 1. Introduction

Rheumatoid arthritis (RA) is a systemic inflammatory autoimmune disease, whereby, in addition to joint damage, patients demonstrate changes to body composition, which includes a reduction in skeletal muscle, with or without the increase in fat mass (termed rheumatoid cachexia or rheumatoid sarcopenia) [1, 2]. Rheumatoid cachexia and rheumatoid sarcopenia are terms often used interchangeably, but while sarcopenia is normally used when referring to the loss of muscle mass and function in the context of aging, cachexia-associated loss is usually associated with underlying disease [1]. Therefore, in this article, the loss of muscle mass will be referred to as rheumatoid cachexia. Regulation of skeletal muscle is complex, with the involvement of numerous cell types and growth factors [3]. The skeletal muscle contains stem cells (termed satellite cells), which become activated, proliferate, differentiate, and fuse to form new myofibers [4]. Among the growth factors involved, hepatocyte growth factor (HGF) stands out as an activator of SCs [5] and the modulation of the myostatin/follistatin axis regulates atrophy [6]. Muscle cells adhere to the extracellular matrix (ECM), a vital component for development, functioning, and signalling within the muscle. Although muscle cells themselves also secrete numerous ECM components, the main contributor to ECM formation is the fibroblasts [7]. This process is largely regulated by transforming growth factor-*β* (TGF-*β*), a protein secreted by fibroblasts, myoblasts, and macrophages. Macrophages, the third cell type, are resident in the connective tissue surrounding the myofibers [8]. Upon injury/insult, these and other circulatory macrophages will be recruited to the site in order to contribute to the degeneration and regeneration process [9]. Pro- and anti-inflammatory macrophages are vital in different stages of the regenerative process, and the shift in macrophage phenotype from M1 to the tissue remodelling M2 phenotype is vital in repair [10]. However, during chronic inflammation, as demonstrated in RA, the balance between M1 and M2, and specifically the persisting presence of M2b macrophages, disrupts the delicate balance between catabolism and anabolism, and the proliferation and differentiation of satellite cells, ultimately resulting in muscle wasting [3]. Employing an *in vitro* model using multiple human cell types to more accurately simulate the cellular niche and cellular responses may prove beneficial in further understanding interactions in both a healthy and diseased muscle environment.

A particular benefit of such a coculture model is the ability to employ intervention treatments to further probe signalling responses or to identify potential therapeutic or preventative modalities. We have selected bone morphogenetic protein-7 (BMP-7)—which belongs to the TGF-*β* superfamily and which is structurally related to growth and differentiation factors [11, 12]—as a potential intervention agent. BMP-7 was initially studied due to its involvement in osteoblast differentiation and bone formation. However, several BMPs exhibit multiple biological activities in different cell types [13]. Overall, research into the role of BMP-7 in RA is limited: while a few studies reported on its role in reducing joint destruction [14, 15], no studies are available in the context of targeting rheumatoid cachexia. Based on the fact that the TGF-*β* signalling network functions as a major component in developing skeletal muscle tissue, it is likely that the BMP axis may also play a pivotal role in muscle mass regulation. Indeed, the injection of BMP-7 vectors into mouse muscle was reported to result in increased myofiber area and diameter [16]. BMP-7 has also been implicated in the resolution of inflammation [17]. Furthermore, in a mouse model of renal fibrosis, BMP-7 treatment reduced the severity of fibrosis and reversed renal pathology [18]. Similarly, RA synovial fluid-stimulated fibroblast-like synoviocytes (FLS) treated with BMP-7 exhibited inhibited production of *α*-SMA, a marker expressed on synovial lining myofibroblasts [14]. Lastly, in a model of zymosan-induced arthritis (ZIA), direct injection with BMP-7 into the affected knee inhibited the loss of cartilage matrix and reduced swelling, as well as attenuating cellular infiltration, reducing IL-1*β* and increasing IL-10 levels [15].

In the current study, we developed a novel triple coculture model using primary muscle fibroblasts, myoblasts, and blood-derived polarised M1 macrophages collected from healthy human donors. These standardised cultures were then exposed to serum from healthy or RA patients to better understand the interactions of various cell types and molecular role players in the muscle environment under conditions of diseased systemic signalling. Secondly, we report on the capacity of BMP-7 to alter the responses of relevant cytokines and growth factors in rheumatoid cachexia. The overall aim of the study was to develop a triple coculture model that—despite some unavoidable limitations—is more patient-specific, allowing the individualised assessment of sensitivity to treatment interventions.

## 2. Methods

### 2.1. Ethics Statement

Ethical clearance for this study was obtained from the Stellenbosch University Health Research Ethics Committee (HREC) for the isolation of myoblasts and fibroblasts from healthy volunteers (reference N12/08/051) and the collection of blood from healthy and RA patients (reference HREC2-2020-13147). Biosafety clearance for the handling of BSL2 samples was obtained from the Biosafety and Environmental Ethics Committee at Stellenbosch University (reference REC:BEE:2020-18524).

### 2.2. Participant Recruitment for Primary Cell Isolation

For the isolation of myoblasts and fibroblasts, muscle biopsies were obtained from the *vastus lateralis* muscle of healthy, normally active young participants who were neither diabetic nor using anti-inflammatory medication, and who did not have recent muscle injury. For the isolation of primary monocytes, blood was obtained in EDTA-coated tubes from healthy, young participants who did not have chronic/acute infections or injuries, smoked, or used anti-inflammatory medication.

### 2.3. Participant Recruitment for Rheumatoid Arthritis Study

Predicting the response of patients to treatment has shown low success rates with a number of patients not responding to treatment, developing resistance or treatment-related adverse events [19, 20]. Blood was obtained in EDTA-coated and SST tubes from RA patients and healthy participants based on the following criteria: RA patients that either were (1) treatment-naïve (RATN) or (2) had moderate to severe, treatment-nonresponding RA (RATNR) were recruited from Winelands Rheumatology Centre, Stellenbosch, South Africa. Patients did not have additional acute/chronic infections, comorbidities, juvenile onset RA, or obesity. RATN patients were recently clinically diagnosed with ongoing active rheumatoid arthritis and were not yet being treated with disease-modifying anti-rheumatic drugs (DMARDs). RATNR patients were compliant on synthetic or biologic DMARDs, but presenting with ongoing disease activity based on clinical signs of active synovitis, suggesting the failure of mono- or poly-pharmacy DMARD therapy. No clear differences in either CRP or ESR values (Supplementary data, Table 2) were evident between groups, but as data were somewhat incomplete, the main classification was assessment (longitudinal in the case of RATNR) of clinical signs of active synovitis by an experienced rheumatologist. Healthy participants (non-RA control, NC) were age-matched and excluded according to the same criteria as above, with the addition of the use of anti-inflammatory medication as an exclusion criterion in the healthy group. Six participants/patients were recruited per group.

### 2.4. Patient/Participant Plasma Analysis

RA patients' and healthy participants' plasma was collected in EDTA tubes and centrifuged at 400 × *g* for 10 minutes at room temperature. Plasma was analysed using the MILLIPLEX human cytokine magnetic bead panel carried out according to the manufacturer's instructions. The following analytes were assessed: TNF-*α*, IL-1*β*, IL-1RA, IL-6, and IL-10 (HCYTOMAG-60K; Merck Millipore, Darmstadt, Germany).

### 2.5. Primary Cell Isolations

Muscle biopsy tissue was obtained from the *vastus lateralis* muscle of normally healthy male volunteers not using any medication, under sterile conditions using a 5-mm trephine biopsy needle (Bergstrom 6 biopsy needle, STILLE, Sweden) with assisted suction, following standard procedures [21]. Biopsy tissue was immediately placed in cold phosphate-buffered saline (PBS; P4417, Sigma-Aldrich) with 10% (v/v) penicillin/streptomycin (15070063, Gibco™) and 1% (v/v) gentamycin. Primary fibroblasts were isolated according to the previously established consecutive preplating protocol [22] within one hour of obtaining samples. Briefly, biopsy tissue was digested in collagenase/dispase solution (10269638001, Sigma-Aldrich) and placed in ECL-precoated flasks. After allowing primary fibroblast attachment for 1 hour, media with unattached cells was removed was discarded. Primary myoblasts were isolated according to the micro-explant technique [21], in which pieces of muscle biopsy were plated on entactin-collagen IV-laminin (ECL; 08-110, Merck, USA)-precoated plates and myoblasts were allowed to migrate out of the tissue. Cells from the third and fourth subculture were used, as initial cells were a combination of myoblasts and fibroblasts.

Prepared pure isolates of primary fibroblasts and myoblasts were cultured in complete Hams-F10 media (N6908, Sigma-Aldrich) supplemented with 20% foetal bovine serum (FBS; 10499-044, Life Technologies), 1% penicillin-streptomycin (P43333, Sigma-Aldrich), and 2.5 ng/ml human recombinant fibroblast growth factor (hrFGF; G5071, Promega). After sufficient stocks were created, primary myoblast and fibroblast media were converted to RPMI 1640 media (with GlutaMAX; 61870010, Gibco) for consistency between the three cell types.

Primary monocytes were isolated from donated blood using a double gradient centrifugation protocol [23]. Monocytes were cultured in RPMI 1640 media containing 20% FBS and 1% penicillin-streptomycin in 24-well plates precoated with ECL. Cells were supplemented with 50 ng/ml of granulocyte macrophage colony-stimulating factor (GM-CSF; SRP3050, Sigma-Aldrich) to allow predifferentiation to occur. Cells were allowed to adhere for 24 hours before the first media change; thereafter, media were changed every 2 days (for 4 additional days). Cells were polarised to a M1 phenotype with 50 ng/ml GM-CSF, 50 ng/ml lipopolysaccharide (LPS; L2762, Sigma-Aldrich), and 20 ng/ml interferon-*γ* (IFN-*γ*; I3265, Sigma-Aldrich) for 24 hours.

### 2.6. Cell Phenotype Confirmation

**Figure 1:**
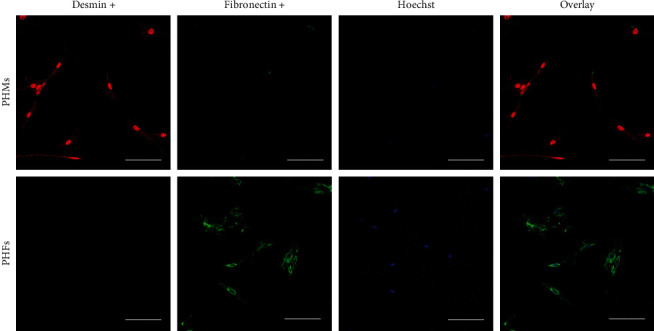
Cell type confirmation of primary human myoblasts (PHMs) and fibroblasts (PHFs) staining with desmin (red) and fibronectin (green), respectively. Nuclei are visualised using Hoechst (blue). Images were captured at 200x magnification. The scale bar represents 100 *μ*m.

To confirm primary human myoblast (PHM) and primary human fibroblast (PHF) phenotype and culture purity, cells were fixed with 4% PFA, blocked, and stained overnight with desmin (ab15200, Abcam, UK) and fibronectin (sc80982, Santa Cruz, USA) at 4°C. Cells were then stained with fluorescence-labelled secondary antibodies (594–150064 and 488–150109, Abcam, UK) and Hoechst (ab33342 Abcam, UK), mounted with fluorescent mounting media (53023, DAKO, Denmark), and imaged on the Zeiss confocal microscope (Carl Zeiss LSM 780, Zeiss, Germany) at 200x magnification. PHM phenotype was confirmed by positive staining with desmin only, while PHF phenotype was confirmed by positive staining with fibronectin only (Figure 1).

### 2.7. Triple Coculture with Patient Serum

Patient serum was collected in SST tubes and after allowing clotting for 30 minutes at room temperature centrifuged at 1500 × *g* for 10 minutes. Media (RPMI 1640) was prepared with 20% patient serum and 1% penicillin-streptomycin. Primary myoblasts and fibroblasts were detached with trypsin (25200072, Gibco). Primary M1-polarised macrophages were detached with Accutase® (A6964, Sigma-Aldrich). Cells were plated on ECL-precoated plates in the patient serum-containing media in the ratio of 40 000 macrophages:10 000 myoblasts:5 000 fibroblasts as determined with intramuscular cell staining in a rodent collagen-induced arthritis model [24]. (The number of macrophages was modified to twice the number present in RA rodents, to correct for known lack of proliferation capacity of the terminally differentiated macrophages over the course of the culture protocol.) Plates were shaken every 15 minutes over a 90-minute period to allow even distribution of cells. Triple cocultures were prepared in duplicate for each patient and treatment condition.

### 2.8. BMP-7 Treatment of Various Cell Types

After 48 hours of exposure of triple cocultures to patient serum-conditioned media, media were replaced with serum-conditioned media and cells treated with 750 ng/ml BMP-7 (prepared in dH_2_O) for an additional 48 hours. The dose of 750 ng/ml was determined in a pilot dose-response study using single cell cultures for all cell types (refer to Figures A1–A4, Supplementary data). After 48 hours, cell culture supernatants were removed and centrifuged at 500 × *g* for 5 minutes to remove remaining cells and debris. Images were taken on the Olympus microscope (CKX41, Olympus Corporation) on day 2 and day 4 at 40x and 100x magnification. In addition, 100x images were analysed using ImageJ software to measure the area fraction of cells (measure of confluence) within each field of view.

### 2.9. Supernatant Analysis

Triple coculture clarified supernatants were analysed using ELISA and Multiplex Quantikine analyses as follows: follistatin (DFN00, R&D Systems), GDF-8/Myostatin (DGDF80, R&D Systems), decorin (NBP3-08102, Novus Biologicals), Fibronectin (E-EL-H0179-96T, E-Lab Bioscience), collagen IV (E-EL-H0178-96T, E-Lab Bioscience), TGF-*β* magnetic Luminex (FCSTM17-01, R&D Systems), and magnetic Luminex for collagen I alpha 1, HGF, IL-1*β*, IL-6, IL-10, and TNF-*α* (LXSAHM-07, R&D Systems).

### 2.10. Statistical Analysis

Statistical analysis was performed on GraphPad Prism v.8. Patient data and plasma results were assessed for normality using Shapiro–Wilk analysis. Data were analysed using a one-way ANOVA and Tukey's multiple comparisons for parametric data, and the Kruskal–Wallis test with Dunn's multiple comparisons for nonparametric data. Correlations were performed using Pearson's correlation.

## 3. Results

### 3.1. Patient Group Characterisation Plasma Cytokine Profiles

As expected, patient groups exhibited variable and different duration of diagnosis periods, with treatment naïve patients reporting having RA for 2.33 ± 3.67 years (four out of six were recently diagnosed), and treatment-nonresponding patients having RA for 11.80 ± 13.33 years. Affected joints included wrists, hands, elbows, ankles, and knees in both groups. Age and body composition did not differ significantly between groups: healthy controls (NC) were 50.2 ± 8.5 yr old (three males, three females; BMI: 29.79 ± 4.57), treatment-naïve (RATN) patients were 53.7 ± 18.9 yr old (one male, five females; BMI: 30.79 ± 7.56), and RA treatment-nonresponding (RATNR) patients were 59.3 ± 14.2 yr old (one male, five female; BMI: 24.89 ± 3.30). Of the recruited patients, four of the six treatment naïve patients stated they had a noticeable loss in muscle mass, whereas two of the six treatment-nonresponding patients confirmed noticeable muscle loss. However, since neither BMI nor subjective reporting of muscle loss correlated with any parameter assessed, these measures are in our opinion not suitable indicators of cachexia progression. Given the cross-sectional design of the current study, no patients were assessed in a quantitative manner for muscle loss over the period of active disease. In order to assess cachexia accurately, a longitudinal study with accurate dual-energy X-ray absorptiometry (DEXA) is probably required.

**Figure 2:**
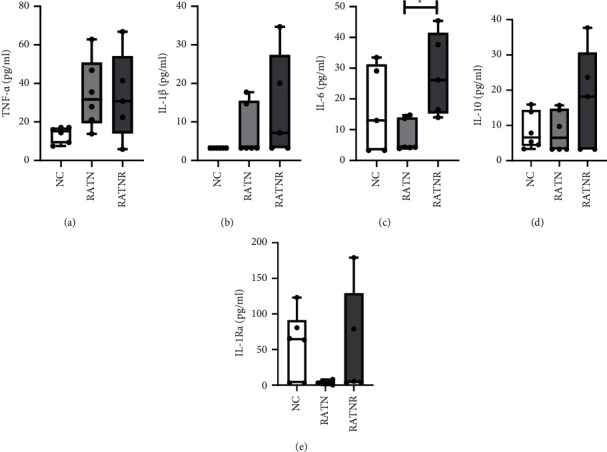
Patient plasma cytokine concentration in healthy (NC), RA treatment naïve (RATN), and RA treatment-nonresponding (RATNR) patients. (a) TNF-*α*; (b) IL-1*β*; (c) IL-6; (d) IL-10; and (e) IL-1Ra. Statistical analysis: one-way ANOVA. ^*∗*^=*p* < 0.05. *n* = 6 per group. Data represented as box and whisker plots indicating the highest and lowest values, and the median and the interquartile range, as well as individual data points. TNF-*α* = tumour necrosis factor-*α*; IL = interleukin.

Comparison of groups for patient plasma cytokines indicated limited number of differences reaching statistical significance, likely due to large variability among patients and a small sample size (Figure 2). Nevertheless, the general picture in both RA groups was in line with a relatively more pro-inflammatory state. In addition, RATNR patients had a significantly increased IL-6 plasma concentration (*p* < 0.05) compared to RATN patients (Figure 2(c)).

**Figure 3:**
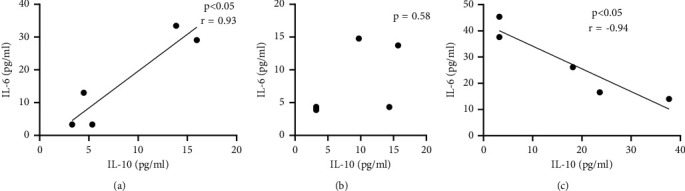
Correlation between IL-10 and IL-6 in the different patient groups. (a) Nonarthritic control (NC); (b) RA treatment naïve (RATN); (c) RA treatment-nonresponding (RATNR). Statistical analysis: Pearson's correlation. *n* = 5 per group.

Correlation between IL-10 and IL-6 demonstrated a statistically significant positive correlation in the NC group that was lost in the RATN group, whereas the RATNR group demonstrated a statistically significant negative correlation (Figure 3).

### 3.2. Triple Coculture Responses

**Figure 4:**
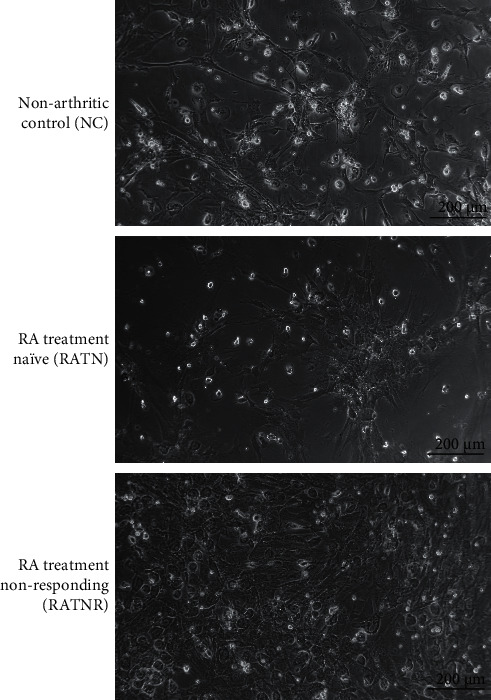
Representative images of the triple cell culture model indicating examples of the different observed responses to the patient serum. Images taken at 100x magnification. The scale bar represents 200 *μ*m.

Qualitatively, healthy participants' cells exhibited a unique response to the serums from different patient groups, as indicated by the representative images in Figure 4. Cells appeared to proliferate at different rates, as is evident from the differences in relative confluence—RATNR serum resulted in the fastest growth rate, resulting in cultures appearing fully confluent after 2 × 48 hours. Macrophages in the RATNR-exposed cultures appeared to demonstrate a greater extent of activation compared to both the other groups. In the RATN group, patient serum resulted in clustered growth patterns of cells.

**Figure 5:**
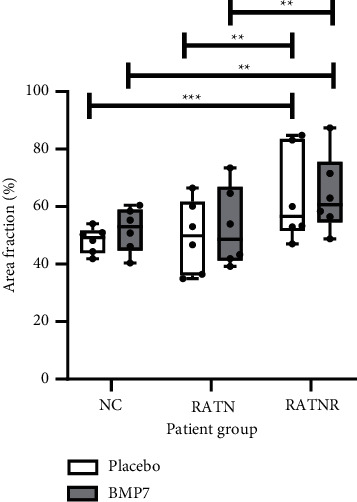
Percentage area fraction taken up by the triple culture cells in the field of view, comparing the cultures exposed to serum from healthy control, treatment-naïve, and treatment-nonresponding RA patients, with or without BMP-7 treatment (750 ng/ml). *n* = 6. Statistical analysis: two-way ANOVA with Tukey's multiple comparisons. ^*∗∗*^=*p* < 0.01; ^*∗∗∗*^=*p* < 0.001. Data represented as box and whisker plots indicating the highest and lowest values, and the median and the interquartile range, as well as individual data points.

Quantification of area fraction (%) as measure of confluence confirmed that cultures treated with RATNR patient serum proliferated more extensively and therefore exhibited a significantly higher area fraction than other patient groups, both with and without BMP-7 treatment (Figure 5).

**Figure 6:**
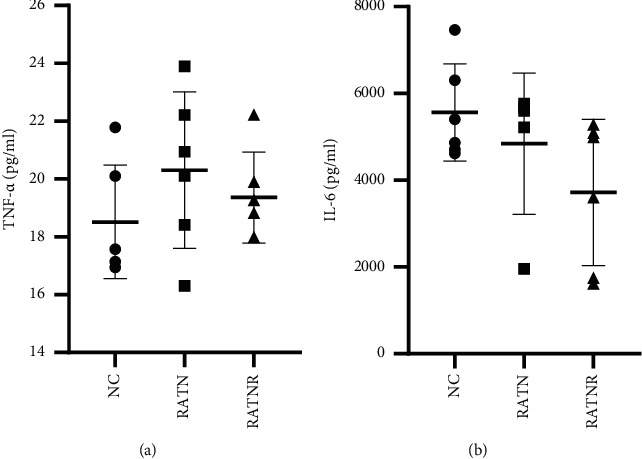
Triple culture supernatant cytokine concentration comparing the conditioned media exposed to healthy (NC), RA treatment naïve (RATN), and RA treatment-nonresponding (RATNR) patients' serum. (a) TNF-*α*; (b) IL-6. Statistical analysis: one-way ANOVA. *n* = 6 per group. Data represented as mean ± SD. TNF-*α* = tumour necrosis factor-*α*; IL = interleukin.

Luminex analysis of culture supernatants generally demonstrated very low levels of IL-1*β* and IL-10. IL-10 concentration was below detectable limits in all groups, while IL-1*β* concentration only measurable in one RATN-exposed culture and two RATNR-exposed cultures (data not shown). These data were thus excluded from interpretation. Similarly, low levels of TNF-*α* were detected across all groups. IL-6 was secreted in relatively high quantities, but appeared similar between groups (Figure 6).

**Figure 7:**
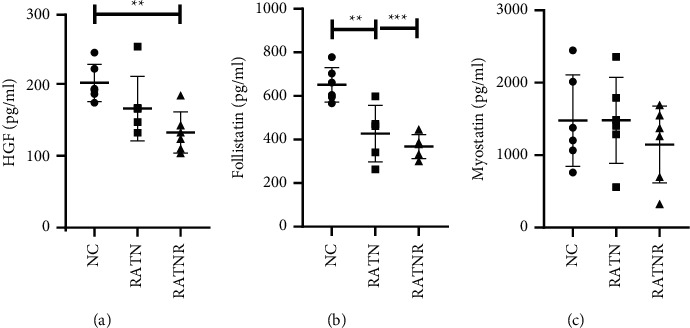
Triple culture supernatant muscle growth factor concentration comparing healthy (NC), RA treatment naïve (RATN), and RA treatment-nonresponding (RATNR) patients. (a) HGF; (b) follistatin; (c) myostatin. Statistical analysis: one-way ANOVA (parametric: c) or Kruskal–Wallis test (nonparametric: (a b). ^*∗∗*^=*p* < 0.01; ^*∗∗∗*^=*p* < 0.001. *n* = 6 per group. Data represented as mean ± SD. HGF = hepatocyte growth factor.

Statistically more significant differences between groups were evident for muscle growth factors (Figure 7). HGF concentration was significantly reduced in the media exposed to RATNR serum when compared to the control, while follistatin concentration was statistically significantly reduced in both the RATN (*p* < 0.01) and RATNR serum-exposed groups (*p* < 0.001) when compared to the control. Myostatin demonstrated no significant differences between the groups.

**Figure 8:**
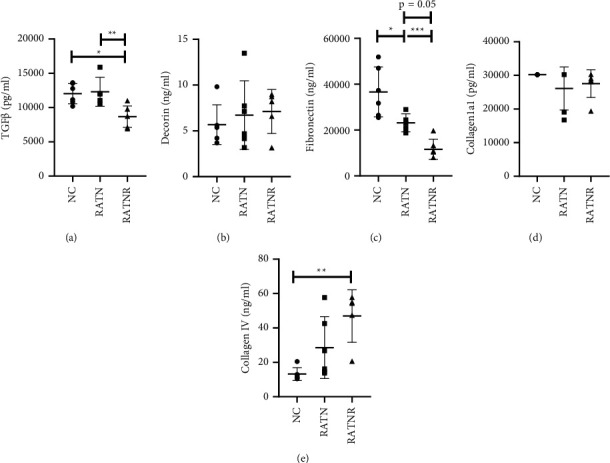
Triple culture supernatant extracellular matrix factor concentrations comparing healthy (NC), RA treatment-naïve (RATN), and RA treatment-nonresponding (RATNR) patients. (a) TGF*β*; (b) decorin; (c) fibronectin; (d) collagen 1a1; (e) collagen IV. Statistical analysis: one-way ANOVA (parametric: (a, b, c, d) or Kruskal–Wallis test (nonparametric: e). ^*∗*^=*p* < 0.05; ^*∗∗*^=*p* < 0.01; ^*∗∗∗*^=*p* < 0.001. *n* = 6 per group. Data represented as mean ± SD. TGF*β* = transforming growth factor-*β*; BMP-7 = bone morphogenetic protein 7.

Turning attention to fibroblast growth factors, TGF*β* concentration was significantly reduced in the RATNR serum-exposed group when compared to both the control group and RATN group (Figure 8(a)). Decorin levels were similar in all groups (Figure 8(b)), while fibronectin concentration was statistically significantly lower than NC in the RATN and RATNR groups, as well as lower (*p*=0.05) in RATNR when compared to RATN (Figure 8(c)). Collagen 1a1 levels were greater than the detectable limit for all NC samples but fell within the detection range for the kit for at least half the samples of the RATN and RATNR groups, suggesting that these groups may exhibit lower collagen 1a1 than the healthy controls (Figure 8(d)). Collagen IV concentration appeared higher in response of both RA serum groups, with a statistically significant increase in the RATNR group compared to healthy controls (Figure 8(e)).

Table 1 presents the effects of BMP-7 on actual concentrations in the NC by providing the placebo and the BMP-7 data. Here, the addition of BMP-7 decreased the concentration of TGF-*β* and fibronectin significantly when the triple culture was cultured in NC serum, suggesting that its main effect is exerted on the fibroblasts at the concentration of 750 ng/ml.

**Figure 9:**
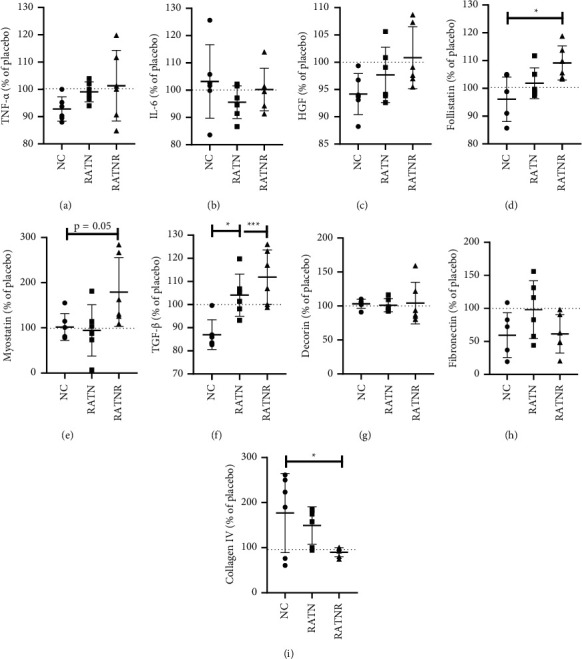
Cellular responses after treatment with BMP-7 expressed as percentage of response in placebo condition, as assessed in triple culture supernatant of a fibroblast, myoblast, and M1 macrophage mixed culture exposed to serum of healthy (NC), RA treatment naïve (RATN), and RA treatment-nonresponding (RATNR) patients. (a) TNF-*α*; (b) IL-6; (c) HGF; (d) follistatin; (e) myostatin; (f) TGF*β*; (g) decorin; (h) fibronectin; (i) collagen IV. Statistical analysis: one-way ANOVA (parametric: a–e, g–i) or Kruskal–Wallis test (nonparametric: f). ^*∗*^=*p* < 0.05; ^*∗∗*^=*p* < 0.01; ^*∗∗∗*^=*p* < 0.001. *n* = 6 per group. Data represented as mean ± SD. TNF-*α* = tumour necrosis factor-*α*; IL = interleukin; HGF = hepatocyte growth factor; TGF*β* = transforming growth factor-*β*; BMP-7 = bone morphogenetic protein 7.

To compare how the culture groups responded differently in the presence of BMP-7, data are presented as a percentage of the placebo condition for each patient, in order to normalise data and maximise measurable effect size. In line with the generally low culture inflammatory cytokine responses, neither TNF-*α* nor IL-6 secretion was significantly affected by BMP-7 treatment (Figures 9(a) and 9(b)). Follistatin concentration, which was significantly decreased as the result of RA serum exposure in the placebo conditions, demonstrated a significant increase in concentration when compared to NC, in the presence of BMP-7 in (Figure 9(d)). A similar normalisation effect of BMP-7 was seen for several other parameters assessed, for example, with myostatin (Figure 9(e)) and TGF*β* concentration (Figure 9(f)), which both exhibited significant increases in the RATNR group and both RA groups, respectively, when compared to the NC group in the presence of BMP-7. There were no significant differences between the patient groups for both decorin (Figure 9(g)) or fibronectin (Figure 9(h)). Collagen IV concentration was increased in the RA group (Figure 8(e)) but significantly decreased in the RATNR group compared to the NC arguably as a result of BMP-7 treatment (Figure 9(i)).

## 4. Discussion

Using a novel triple culture technique to simulate the skeletal muscle niche, current data contribute to our understanding of rheumatoid cachexia mechanisms in RA and RA treatment failure. Furthermore, the efficacy of BMP-7 as potential treatment modality is highlighted.

Before interpreting the effect of a normal mixed culture to patient and control serum, it is necessary to consider differences between the experimental group plasma profiles. In terms of plasma characterisation, cytokine profiles demonstrated the expected interindividual variability in all groups. Nevertheless, both plasma TNF-*α* and IL-1*β* tended to be more variable between individuals—and somewhat higher than those of controls—in both RA groups, which is in line with their known role in the pathogenesis of RA [25, 26]. Similarly, the barely detectable levels of IL-1Ra in RATN patients are again in line with literature correlating this profile with RA disease development [26, 27]. Various treatment options result in the downstream increase in IL-1Ra [28–30], in line with the results demonstrated in the RATNR group. Given its major role as myokine [31], IL-6 was also assessed in the current study. However, given the high variability of this parameter even in the control group, interpretation of IL-6 data in isolation was not informative in the current context. However, when correlated with IL-10 levels, important cytokine dysregulation became evident. Under healthy circumstances, IL-6 results in the upregulation of IL-10 [32, 33]. However, this relationship appears to be dysregulated in the RATNR group, where a negative correlation is observed. This relative failure to upregulate IL-10 in response to IL-6 is in line with our earlier suggestion of a failure of RA macrophages to switch to the anti-inflammatory M2c phenotype, which is responsible for IL-10 release [3] as well as our recent study in CIA rats, which demonstrated decreased IL-10 concentration in muscle to be a robust marker for rheumatoid cachexia in this model [24]. This aspect should be elucidated further in longitudinal studies in human RA patients, to fully evaluate the potential of IL-10 as a biomarker of disease progression and risk of muscle cachexia in particular. Taken together, the plasma profile data confirm that the samples used as stimulus in the triple cultures were indeed representative of the expected control and RA cytokine profiles.

Turning attention to the triple culture data, cell growth patterns between the three serum conditions differed significantly. RATNR serum-conditioned media resulted in increased cellular growth rates when compared to the other groups. However, despite the abundance of cells present in the RATNR serum-exposed group, the myoblasts did not attempt to align and begin the differentiation and fusion process. Additionally, based on their appearance, the macrophages in this group exhibited a greater extent of activation. These effects were likely the result of various growth factors and cytokines present in the serum stimulating the proliferation of myoblasts and fibroblasts. This aligns with the data observed in the rodent CIA model where increased cellular presence was observed; however, the ratio between the cells did not differ [24]. While distinguishing between the cells in single culture was possible due to the low desmin/high fibronectin expression in fibroblasts and low fibronectin/high desmin expression in myoblasts, coculture presents some challenges in this regard. When cultured in close proximity—as they also grow in vivo—myoblasts and fibroblasts interact, resulting in high expression levels for both fibronectin and desmin in both cell types [34–36], making differentiation between them impossible. Therefore, the determination of individual cell type counts was not possible. Perhaps labelling either fibroblasts or myoblasts with GFP prior to coculture could clarify specific cell distribution in future studies. Nevertheless, the most valuable information to be gained from a coculture model pertains to the processes at play when different cell types interact in a tissue niche, such as the changes in nett secretory products in the muscle niche assessed in the current study. Additionally, the assessment of tissue-level signalling is very limited in rheumatoid cachexia research, highlighting the novelty and importance of the current comprehensive data set.

Our interpretation that the relatively pro-inflammatory cytokine profile in the RA patients' plasma may have contributed to the enhanced cellular growth demonstrated in the coculture is in line with literature reporting that TNF-*α*, IL-1*β,* and IL-6 induce myoblast proliferation [37]. Furthermore, the negative IL-6/IL-10 correlation in the RATNR plasma contributes to a dysregulated muscle growth pattern—the magnitude of secreted IL-6 in the triple culture is indicative of a muscle response, rather than an inflammatory response [31]. Different concentrations of IL-6 have differential effects on myoblasts—low concentrations result in proliferation and high concentrations result in differentiation [38]. Despite a higher plasma IL-6 concentration, the relatively lower secreted IL-6 in the RATNR coculture group may be indicative of an altered response to IL-6, thereby affecting the ability to differentiate. Extensive myoblast/satellite cell proliferation and inhibited differentiation is proposed in chronic inflammatory conditions and RA due to the altered inflammatory profile [3]. The same magnitude of secreted TNF-*α* is not observed in the triple culture in general, further confirming that IL-6 secretion is indeed a myoblast response. The presence of relatively low TNF-*α* in the supernatant across all groups indicates that the macrophage response, while present, is not as extensive as that of the myoblasts (for which the IL-6 response is a metabolic and not an inflammatory one [39]). Furthermore, an extensive review reports that muscle and myoblasts have low constitutive expression of TNF-*α* and that responses are better observed in the plasma or serum [40], confirming this interpretation.

In terms of myoblast response, current data illustrate the significant dysregulation of normal muscle maintenance signalling in RA. Normally, the release of nitric oxide (NO) by active skeletal muscle and macrophages would lead to the release of HGF and subsequent satellite cell activation [41–43], while IL-6 promotes the production of HGF [44]. NO also induces the expression of follistatin [41, 42] to contribute to hypertrophy through satellite cell activation, proliferation, and differentiation [45, 46] and in rodents demonstrates improved regeneration and reduced fibrosis. Another factor contributing to myogenesis, TGF-*β*, is a multifunctional cytokine exhibiting various effects on different cell types. [47, 48]. The aberrant downregulated tendency of IL-6, HGF, follistatin, and TGF-*β* in the RATNR serum-exposed group suggests that rheumatoid cachexia may, at least in part, result from a balance-shift to favour the proliferation of muscle tissue, while failing to allow for sufficient differentiation of newly formed cells. In further support of this interpretation, in epithelial cells, TGF*-β* has been reported to repress the expression of the inhibitor of differentiation (Id) family, including Id2 [49]. The reduction in TGF-*β* observed in this study may thus also have resulted in an increase in Id2, contributing to inhibition of differentiation. This is in agreement with a previous rodent collagen-induced arthritis study by our group, where increased Id2 was indeed observed [24]. This provides additional insight into the occurrence of rheumatoid cachexia despite an increase in myogenic regulatory factors, including myogenin, as reported by our group and others [50].

**Figure 10:**
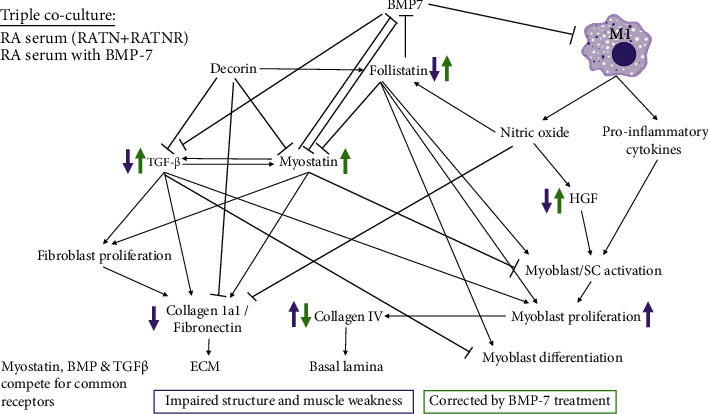
Summary of pathways implicated in the triple coculture experiment as proposed by literature. The purple arrows indicate the changes occurring as a result of RA patient serum-conditioned media. The green arrows indicated the changes occurring as a result of BMP-7 treatment. BMP-7 = bone morphogenetic protein-7; HGF = hepatocyte growth factor; TGF-*β* = transforming growth factor-*β*.

The extracellular environment is another major contributor to signalling, either to enhance or limit tissue maintenance processes. The muscle fibres reside in a scaffold composed of various structural components, referred to as the extracellular matrix (ECM). The ECM is vital in numerous physiological processes in the regulation of muscle development, growth, and repair through its interactions with various cell types, including fibroblasts and immune cells [51]. For example, TGF-*β* is sequestered to the ECM to upregulate components vital to the structure of the ECM, providing stability and a site for protein interactions [48]. However, TGF-*β* also contributes to fibrosis by stimulating excessive proliferation of fibroblasts and secretion of ECM components, along with the inhibition of degradation enzymes [52]. Of the collagens present in the ECM, types I and III are the most abundant in the ECM, whereas type IV provides a network structure to form the basal lamina [51]. Fibronectin, another ECM component, also influences the balance between differentiation and self-renewal, ultimately maintaining the regenerative capacity of the muscle [51, 53]. Both myoblasts and fibroblasts are involved in the production of collagens and fibronectin [54, 55]. Here, the reduced TGF-*β* demonstrated in the RATNR serum-exposed group coincides with the same decreasing trend observed for collagen 1a1 and fibronectin, ultimately indicating impaired structure and organisation. Reduced fibronectin is also already observed in the RATN group, indicating impaired ECM early in disease development [56]. However, collagen IV is increased in the RATNR group. Extensive myoblast proliferation may contribute to the increased collagen IV secretion to form the basal lamina. However, altered basal lamina production as a result of increased collagen IV in aged muscle influences the regulation of satellite cell division resulting in impaired satellite cell numbers [51]. This suboptimal organisation may be one of the causes of the functional deficits observed in RA patients [57, 58]. The effect of RA serum on the triple coculture is summarised in Figure 10.

In terms of intervention, the treatment of the coculture with BMP-7 largely reversed the undesired cellular responses observed after exposure to RA serum and, more specifically, normalised the responses of cells treated with RATNR serum, improving deficits in muscle growth markers and ECM markers, without increasing the deposition of fibronectin, indicating a beneficial role of this treatment.

Furthermore, in the context of macrophage phenotype specifically, pilot data indicate the treatment of primary M1 macrophages with 500 and 750 ng/ml of BMP-7 for 48 hours resulted in the increased presence of M2c macrophages (Figure A1, Supplementary data). This is in line with literature reporting similar effects for BMP-7 in non-RA inflammation models in cells and rodents [12, 17]. In the triple coculture model, this benefit of BMP-7 was not evident from the measured cytokine profile. However, this may have been the effect of relative overgrowth of myoblasts and fibroblasts while macrophages don't proliferate. Although increasing the proportion of macrophages in this culture even further may allow for a more representative picture of the macrophage signalling, the beneficial effect of BMP-7 on overall signalling supports a shift to an anti-inflammatory phenotype. Given the known lingering presence of M1 and M2b macrophages in RA [3], current data in single and coculture warrant further investigation of BMP-7 as a treatment modality in RA.

When addressing muscle growth changes, fibre hypertrophy, as observed following the injection of BMP-7 vectors into healthy mouse muscle [16], would be beneficial to RA patients. Preliminary data indicated that when treating primary myoblasts with different dosages of BMP-7, 750 ng/ml resulted in a greater myoblast size (Figures A2 and A3, Supplementary data). The reversal of both HGF and follistatin effects in the RATNR serum-exposed cultures highlights a potentially beneficial effect. One conflicting factor that may inhibit this effect is the increased myostatin in the RATNR serum-exposed group, due to its ability to bind to the BMP-7 receptors [59], thereafter inhibiting its effects.

Lastly, based on the increased presence of fibrosis in rheumatoid cachexia [60] and the above findings of impaired ECM formation in RATNR serum-exposed cocultures, BMP-7 altering the ECM challenges would be beneficial. As observed in the NC serum-exposed group, BMP-7 has antifibrotic effects through the inhibition of TGF-*β* [61] and reduces the accumulation of ECM. This could also be through enhanced ECM degradation as a result of matrix metalloproteinase (MMP) activity [62, 63]. While the opposite is observed with the RATNR group, this effect is still the reversal of the outcomes observed with RATNR serum alone, thereby improving the overall structure of the ECM and improving the overall outcome. The effect of BMP-7 treatment on RA patient-exposed coculture is summarised in Figure 10.

## 5. Conclusion

Current data demonstrated plasma cytokine differences indicative of healthy controls and RA patients, with a more severe outcome in treatment-nonresponsive patients, which may be either due to treatment resistance itself, or a longer duration of disease progression when compared to treatment naïve patients. One of the key factors in the RATNR group is the dysregulation between plasma IL-6 and IL-10, which may impact the downstream muscle effects. Through the use of a novel, primary triple coculture method, the RATNR serum-exposed group exhibited an extensive capacity for cell growth, despite downregulated HGF and follistatin, and suboptimal ECM organisation when compared to controls. BMP-7 treatment showed beneficial results by reversing the aberrant tendencies observed in the cultures exposed to RA serum.

On a practical note, current data generated in response to patient serum highlight the complexities faced when interpreting data combining multicell culture with the *in vivo* “cocktail” of circulating parameters. However, in our opinion, such comprehensive, complex investigations are required in order to understand or address conditions such as RA and RA cachexia, as these present as equally complex problems that cannot be accurately simulated in simplified protocols. In support of this, current data illustrate our novel coculture model to be an accurate simulation of signalling events in RA capable of reflecting treatment resistance and thus potentially a powerful tool in understanding rheumatoid cachexia and developing patient-specific treatment strategies.

## Figures and Tables

**Table 1:** Comparison of the concentrations of cytokines and growth factors in the triple coculture exposed to NC patient serum after being treated with placebo or BMP-7. Data represented as mean ± SD. ^*∗*^=*p* < 0.05. *n* = 6 per group. TNF-*α* = tumour necrosis factor-*α*; IL = interleukin; HGF = hepatocyte growth factor; TGF-*β* = transforming growth factor-*β*.

Treatment	Nonarthritic control (NC)	
	Placebo	BMP-7
TNF-*α* (pg/ml)	18.52 ± 2.0	17.15 ± 1.5
IL-6 (pg/ml)	5561 ± 1122.8	5710 ± 1227.4
HGF (pg/ml)	201.6 ± 25.8	189.4 ± 20.7
Follistatin (pg/ml)	651.3 ± 79.3	625.2 ± 84.9
Myostatin (pg/ml)	1478 ± 631.0	1381 ± 319.5
TGF-*β* (pg/ml)	12017 ± 1478	10423 ± 1198^*∗*^
Decorin (ng/ml)	5.68 ± 2.17	5.90 ± 2.38
Fibronectin (ng/ml)	36633 ± 10883	19408 ± 7173^*∗*^
Collagen IV (ng/ml)	13.20 ± 3.71	21.89 ± 9.30

## Data Availability

All data used to support the findings of this study are included within the article or provided as supplementary materials.

## Supplementary Materials

Supplementary Materials Supplementary file 1: data present the dose-dependent effects of BMP-7 in single cultures of primary macrophages, myoblasts, and fibroblasts, which were used to optimise BMP-7 treatment dose for the triple culture experiment. In addition, more detailed patient characteristics is provided.
