# Supplementary material for: Therapeutic Benefit in Rheumatoid Cachexia Illustrated Using a Novel Primary Human Triple Cell Coculture Model
Source: Int J Inflam. 2022 Jun 2;2022:1524913. doi: 10.1155/2022/1524913 (PMC9184217; doi:10.1155/2022/1524913)
Supplement: Supplementary Materials — Supplementary file 1: data present the dose-dependent effects of BMP-7 in single cultures of primary macrophages, myoblasts, and fibroblasts, which were used to optimise BMP-7 treatment dose for the triple culture experiment. In addition, more detailed patient characteristics is provided. [file 1524913.f1.docx]

# Supplementary data

Table 2: Table indicating patient data including disease characteristics and current treatment. RATN = rheumatoid arthritis treatment naïve; RATNR = rheumatoid arthritis treatment non-responding; NC = non-arthritis control; BMI = body mass index; CRP = C-reactive protein; ESR = erythrocyte sedimentation rate.

| \|  \| **Age** \| **Weight (kg)** \| **Height (cm)** \| **BMI** \| **Ethnicity** \| **Gender** \| **Affected joint** \| **Diagnosed** \| **Treatment** \| **CRP (mg/L)** \| **ESR (mm/hr)** \| **Muscle Loss** \| \| --- \| --- \| --- \| --- \| --- \| --- \| --- \| --- \| --- \| --- \| --- \| --- \| --- \| \| **RATN1** \| 40 \| 69,2 \| 161 \| 26,70 \| Coloured \| Female \| Wrists, ankles \| Recent \| None \| - \| 75 \| Y \| \| **RATN2** \| 56 \| 72 \| 148 \| 32,87 \| Coloured \| Female \| R. Hand \| Recent \| None \| - \| 34 \| Y \| \| **RATN3** \| 84 \| 72 \| 175 \| 23,51 \| White \| Male \| R. Hand \| Recent \| None \| 0,6 \| - \| - \| \| **RATN4** \| 30 \| 77,9 \| 155 \| 32,42 \| Coloured \| Female \| Elbows, hands, knees, ankles \| 6 years \| None \| - \| 60 \| Y \| \| **RATN5** \| 63 \| 76 \| 180 \| 23,46 \| White \| Female \| All joints \| Recent \| None \| 16,3 \| - \| - \| \| **RATN6** \| 49 \| 87 \| 167 \| 31,20 \| White \| Female \| Knees, wrist, fingers \| 8 years \| None \| 6 \| - \| Y \| \| **RATNR1** \| 70 \| 65,5 \| 168 \| 23,49 \| White \| Female \| Small joints \| 9 years \| Methotrexate, Prednisone \| - \| 12 \| N \| \| **RATNR2** \| 40 \| 52 \| 157 \| 21,10 \| White \| Female \| Left wrist \| - \| Methotrexate, Prednisone \| 1,9 \| - \| N \| \| **RATNR3** \| 68 \| 70 \| 163 \| 26,35 \| White \| Female \| All joints \| 8 years \| Salazopyrin, Prednisone \| 63,6 \| - \| - \| \| **RATNR4** \| 74 \| 74,8 \| - \| - \| White \| Female \| - \| 6 years \| Actemra, Chloroquine \| 0,6 \| - \| Y \| \| **RATNR5** \| 44 \| - \| - \| - \| Coloured \| Male \| Hands, elbows, feet, knees \| 1 year \| Methotrexate, Prednisone, Salazopyrin \| 3,9 \| - \| N \| \| **RATNR6** \| 60 \| 78 \| 165 \| 28,65 \| White \| Female \| Hands, feet, ankles, wrists \| 35 years \| Humira \| 19 \| - \| Y \| \| **NC1** \| 48 \| 95 \| 175 \| 31,02 \| White \| Female \| N/A \| N/A \| N/A \| NA \| NA \| - \| \| **NC2** \| 54 \| - \| - \| - \| White \| Female \| N/A \| N/A \| N/A \| NA \| NA \| - \| \| **NC3** \| 37 \| 67 \| 163 \| 25,22 \| Coloured \| Female \| N/A \| N/A \| N/A \| NA \| NA \| - \| \| **NC4** \| 62 \| 86 \| 185 \| 25,13 \| White \| Male \| N/A \| N/A \| N/A \| NA \| NA \| - \| \| **NC5** \| 46 \| 120 \| 194 \| 31,88 \| White \| Male \| N/A \| N/A \| N/A \| NA \| NA \| - \| \| **NC6** \| 54 \| 140 \| 198 \| 35,71 \| White \| Male \| N/A \| N/A \| N/A \| NA \| NA \| - \| |  |  |  |  |  |  |  |  |  |
| --- | --- | --- | --- | --- | --- | --- | --- | --- | --- | --- | --- | --- | --- | --- | --- | --- | --- | --- | --- | --- | --- | --- | --- | --- | --- | --- | --- | --- | --- | --- | --- | --- | --- | --- | --- | --- | --- | --- | --- | --- | --- | --- | --- | --- | --- | --- | --- | --- | --- | --- | --- | --- | --- | --- | --- | --- | --- | --- | --- | --- | --- | --- | --- | --- | --- | --- | --- | --- | --- | --- | --- | --- | --- | --- | --- | --- | --- | --- | --- | --- | --- | --- | --- | --- | --- | --- | --- | --- | --- | --- | --- | --- | --- | --- | --- | --- | --- | --- | --- | --- | --- | --- | --- | --- | --- | --- | --- | --- | --- | --- | --- | --- | --- | --- | --- | --- | --- | --- | --- | --- | --- | --- | --- | --- | --- | --- | --- | --- | --- | --- | --- | --- | --- | --- | --- | --- | --- | --- | --- | --- | --- | --- | --- | --- | --- | --- | --- | --- | --- | --- | --- | --- | --- | --- | --- | --- | --- | --- | --- | --- | --- | --- | --- | --- | --- | --- | --- | --- | --- | --- | --- | --- | --- | --- | --- | --- | --- | --- | --- | --- | --- | --- | --- | --- | --- | --- | --- | --- | --- | --- | --- | --- | --- | --- | --- | --- | --- | --- | --- | --- | --- | --- | --- | --- | --- | --- | --- | --- | --- | --- | --- | --- | --- | --- | --- | --- | --- | --- | --- | --- | --- | --- | --- | --- | --- | --- | --- | --- | --- | --- | --- | --- | --- | --- | --- | --- | --- | --- | --- | --- | --- | --- | --- | --- | --- | --- | --- | --- | --- | --- | --- | --- | --- | --- | --- | --- |

**Purpose of the single cell culture experiments**

The aim of the pilot single cell culture experiments were to determine the optimal BMP-7 concentration to alter the inflammatory phenotype from a M1 to a M2 phenotype, to improve muscle growth and proliferation without changing the cellular phenotype to an osteogenic profile, and to reduce fibrosis indicators.

**Methods**

Primary blood-derived monocytes were differentiated and polarised to a M1 phenotype before being treated with incremental concentrations of BMP-7 (0, 50, 250, 500, 750 ng/ml) in FBS-containing media for 48 hours. Flow cytometry was performed to determine the percentage of M2c macrophages in the culture through the identification of extracellular markers (CD14, CD163, CD206, viability) and intracellular markers (IL-10, Arginase-1).

Primary myoblasts were plated in 6-well plates with FBS-containing media and treated with incremental concentrations of BMP-7 for 48 hours. Images were taken on the Olympus bright field microscope at 40x and 100x magnification for the assessment of area fraction and cell size. Protein was extracted for analysis of BMP-7 and its receptor, pSmad1/5/8, and muscle-specific growth factors (TGF-β, MyoD, Pax7, Id2) and Runx-2 (osteoblast marker). Scratch analysis was performed on myoblasts in 24-well plates treated with BMP-7 to determine migration ability.

Primary fibroblasts were plated in 6-well plates with FBS-containing media and treated with incremental concentrations of BMP-7 for 48 hours. Images were taken on the Olympus bright field microscope at 40x and 100x magnification for the assessment of area fraction. Protein was extracted for analysis of BMP-7, pSmad1/5/8, and fibroblast-specific growth factors (TGF-β, Id2, α-SMA).

**Results**

Flow cytometry analysis demonstrated that M1 macrophage monocultures treated with BMP-7 did not reduce the viability of cells (Figure A1a). Flow cytometry of cells stained with CD14 indicated that between 70-80% of cells were successfully differentiated to macrophages from monocytes. There were no significant differences in differentiation success between the treatment dose groups (Figure A1b). Treatment with 500 and 750 ng/ml of BMP-7 significantly increased the percentage of cells polarized to an anti-inflammatory M2c phenotype (p<0.05; Figure A1c), as determined by concurrent positive staining with CD14, CD163, CD206, IL-10 and Arginase-1.


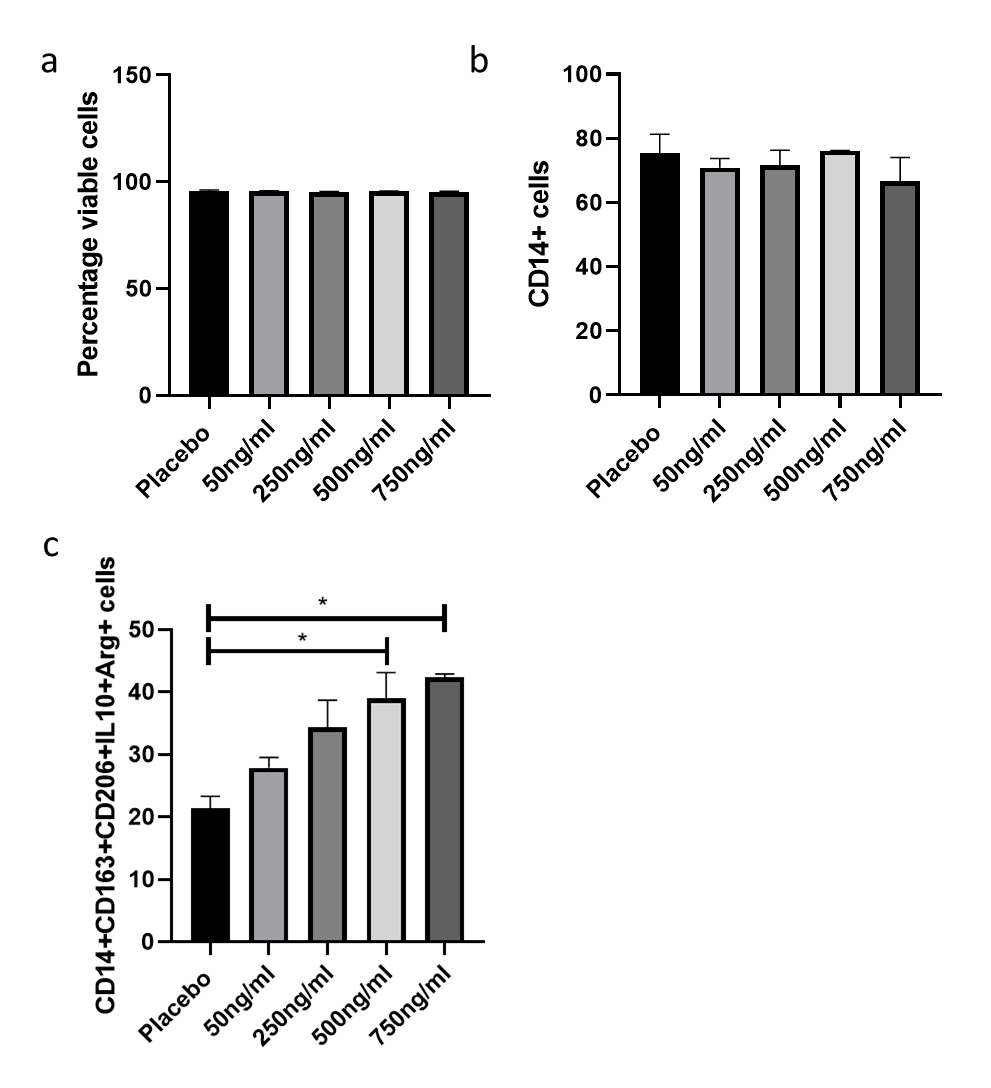


Figure A1: Flow cytometric analysis of M1 macrophages treated with BMP-7 for 48 hours. a) Percentage of viable cells; b) Percentage of CD14+ cells; c) Percentage of M2c macrophages as indicated by positive signal for CD14, CD163, CD206, IL-10 and Arginase-1. n=2 per group. Statistical analysis: One-way ANOVA with Tukey’s multiple comparisons test. * = p<0.05. BMP-7 = bone morphogenic protein-7.

Analysis of myoblast cell images was used to assess area fraction, cell size and migration ability with a scratch assay. Area fraction of the myoblasts did not differ significantly with increasing concentrations of BMP-7 (Figure A2a). However, cell size did increase following treatment with 750 ng/ml of BMP-7 (p<0.05; Figure 2b). There were no significant differences in migration ability between the dosages, except at 1- hour post-scratch where 50 ng/ml demonstrated reduced migration ability (p<0.05; Figure 2c).


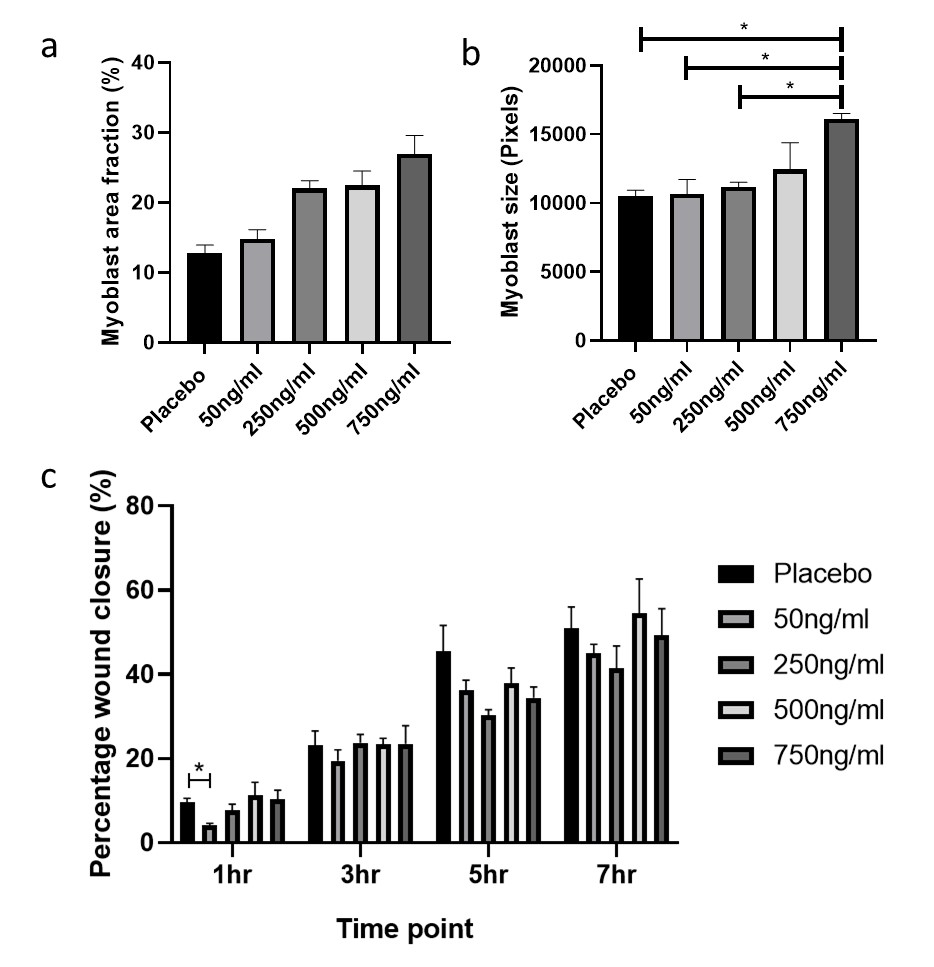


Figure A2: Myoblast response to 48-hour BMP-7 treatment. a) Myoblast area fraction; b) Myoblast cell size; c) Percentage wound closure over 7 hours following scratch assay. n=3 per group. Statistical analysis: one-way ANOVA with Tukey’s multiple comparisons test (a); Kruskal-Wallis test with Dunn’s multiple comparisons test (b); and two-way ANOVA with Tukey’s multiple comparisons test (c). * = p<0.05.

Western blot analysis of myoblasts demonstrated no significant effect of BMP-7 treatment on BMP-7, pSmad1/5/8 receptor, TGF-β, MyoD, and Pax7 concentration (Figure A3a-e). Id2 protein concentration was significantly greater than the placebo following treatment with 250 ng/ml of BMP-7 (p<0.01; Figure A3f). Runx2, a marker of bone differentiation, was significantly lower than day 3 and day 7 differentiating chondroblast controls in the placebo, 50, 250 and 500 ng/ml groups (p<0.05; Figure A3g), indicating no bone differentiation as a result of BMP-7 treatment.


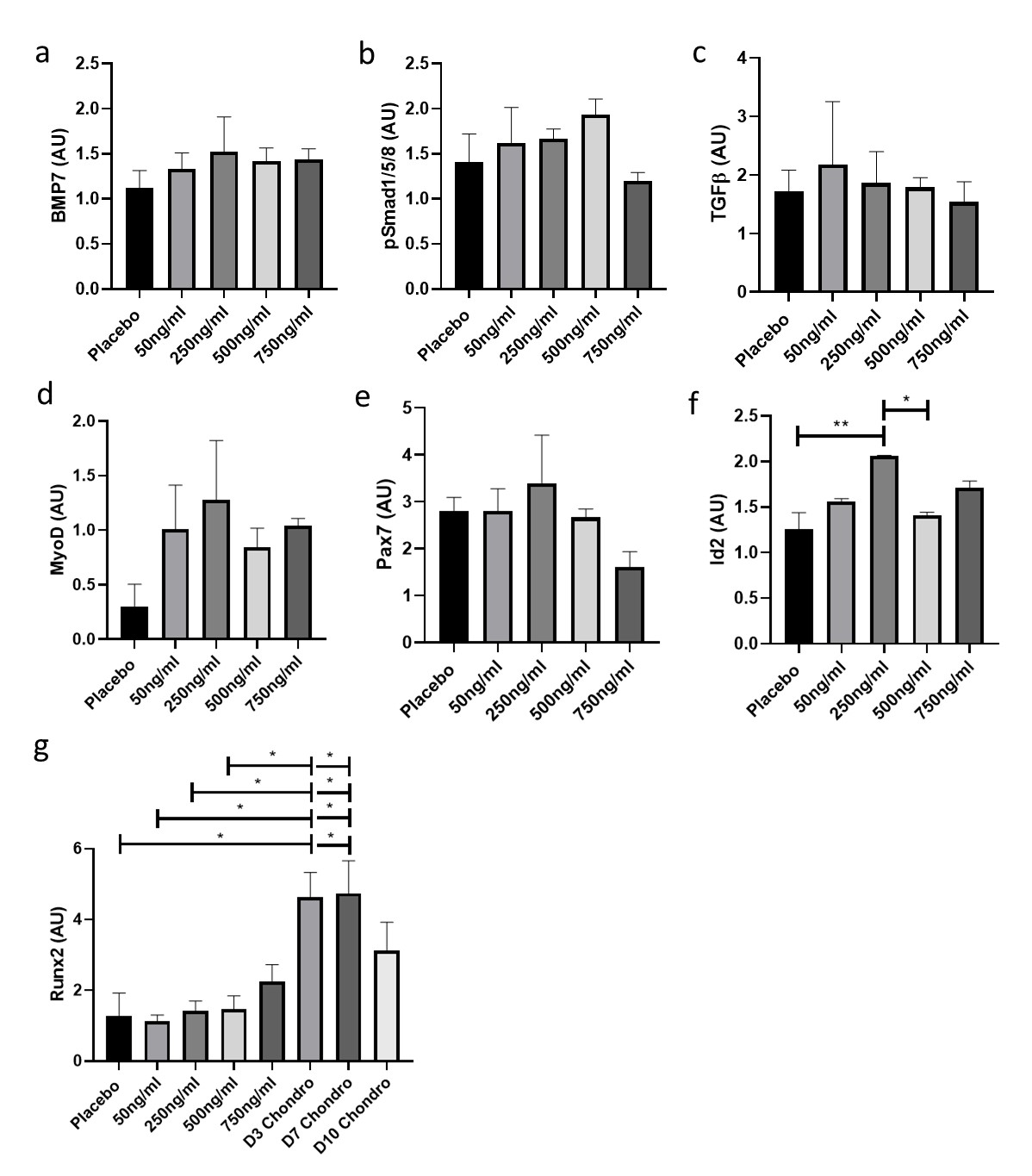


Figure A3: Western blot analysis of myoblast protein following BMP-7 treatment for 48 hours. a) BMP-7; b) pSmad1/5/8; c) TGFβ; d) MyoD; e) Pax7; f) Id2; g) Runx2 (with differentiation chondroblasts as a control). n=3 per group. Statistical analysis: One-way ANOVA with Tukey’s multiple comparisons test. * = p<0.05; ** = p<0.01. BMP-7 = bone morphogenic protein-7; TGFβ = transforming growth factor-β; Pax7 = paired box 7; Id2 = inhibitor of differentiation 2; Runx2 = runt-related transcription factor-2.

Image analysis of fibroblasts indicated a significant difference in area fraction between the 50 ng/ml and 750 ng/ml BMP-7-treated cells (p<0.05; Figure A4a). Western blot analysis demonstrated no significant effect of BMP-7 treatment on BMP-7, pSmad1/5/8, TGFβ, and α-SMA (Figure A4b-d, f). However, Id2 protein concentration was significantly elevated following treatment with 750 ng/ml of BMP-7 (p<0.05; Figure A4e).


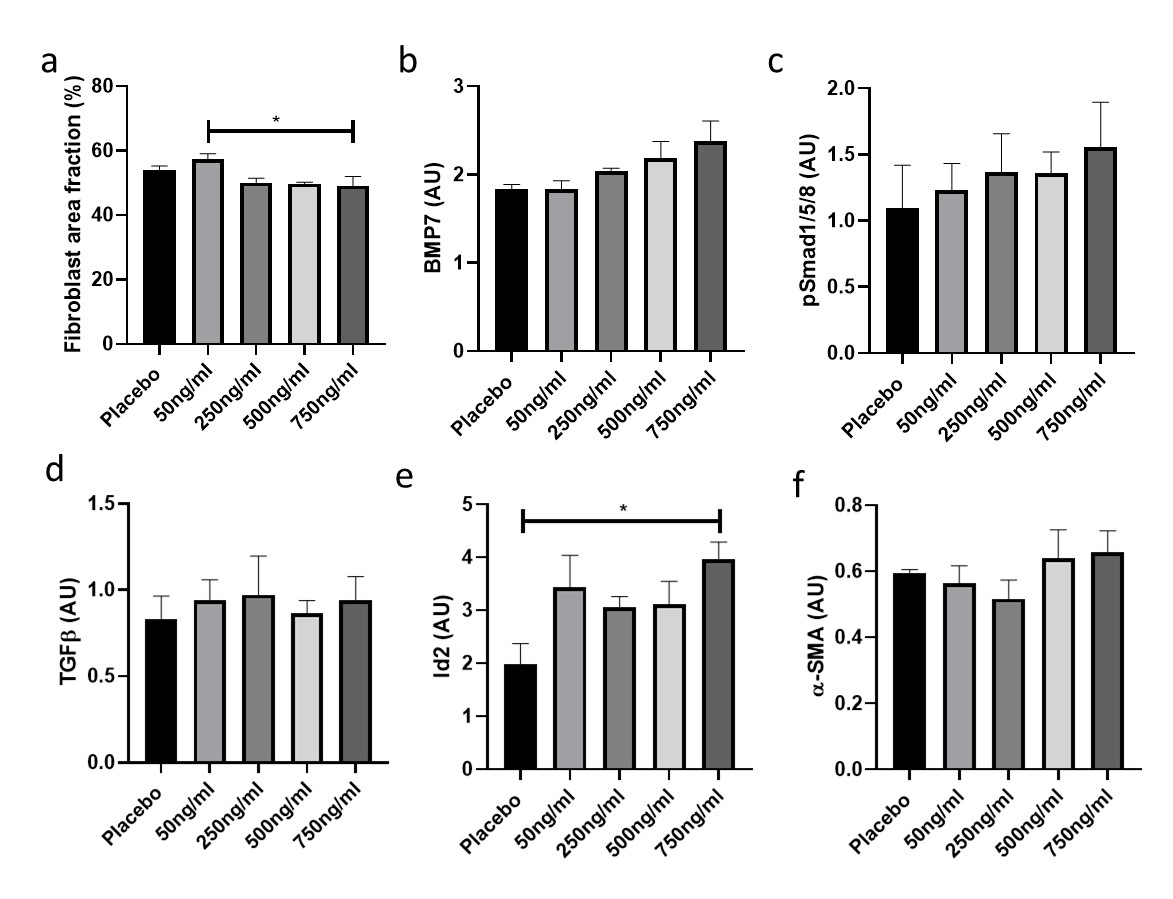


Figure A4: Fibroblast area fraction and Western blot analysis of fibroblast protein following BMP-7 treatment for 48 hours. a) area fraction; b) BMP-7; c) pSmad1/5/8; d) TGFβ; e) Id2; f) α-SMA. n=3 per group. Statistical analysis: One-way ANOVA with Tukey’s multiple comparisons test. * = p<0.05. BMP-7 = bone morphogenic protein-7; TGFβ = transforming growth factor-β; Id2 = inhibitor of differentiation 2; α-SMA = α-smooth muscle actin.

**Conclusion**

The data suggest that the optimal BMP-7 dose to alter macrophages to a beneficial M2 phenotype, improve muscle growth without altering the phenotype to that of osteoblasts, and reduce fibroblast growth is 750 ng/ml.
